# Extracellular Matrix and Growth Factor Engineering for Controlled Angiogenesis in Regenerative Medicine

**DOI:** 10.3389/fbioe.2015.00045

**Published:** 2015-04-01

**Authors:** Mikaël M. Martino, Sime Brkic, Emmanuela Bovo, Maximilian Burger, Dirk J. Schaefer, Thomas Wolff, Lorenz Gürke, Priscilla S. Briquez, Hans M. Larsson, Roberto Gianni-Barrera, Jeffrey A. Hubbell, Andrea Banfi

**Affiliations:** ^1^Host Defense, Immunology Frontier Research Center, Osaka University, Osaka, Japan; ^2^Cell and Gene Therapy, Department of Biomedicine, Basel University, Basel, Switzerland; ^3^Department of Surgery, Basel University Hospital, Basel, Switzerland; ^4^Plastic, Reconstructive, Aesthetic and Hand Surgery, Department of Surgery, Basel University Hospital, Basel, Switzerland; ^5^Vascular Surgery, Department of Surgery, Basel University Hospital, Basel, Switzerland; ^6^Institute of Bioengineering, School of Life Sciences, Ecole Polytechnique Fédérale de Lausanne (EPFL), Lausanne, Switzerland; ^7^Institute for Molecular Engineering, University of Chicago, Chicago, IL, USA; ^8^Argonne National Laboratory, Materials Science Division, Argonne, IL, USA

**Keywords:** angiogenesis, growth factors, extracellular matrix, fibrin, protein engineering

## Abstract

Blood vessel growth plays a key role in regenerative medicine, both to restore blood supply to ischemic tissues and to ensure rapid vascularization of clinical-size tissue-engineered grafts. For example, vascular endothelial growth factor (VEGF) is the master regulator of physiological blood vessel growth and is one of the main molecular targets of therapeutic angiogenesis approaches. However, angiogenesis is a complex process and there is a need to develop rational therapeutic strategies based on a firm understanding of basic vascular biology principles, as evidenced by the disappointing results of initial clinical trials of angiogenic factor delivery. In particular, the spatial localization of angiogenic signals in the extracellular matrix (ECM) is crucial to ensure the proper assembly and maturation of new vascular structures. Here, we discuss the therapeutic implications of matrix interactions of angiogenic factors, with a special emphasis on VEGF, as well as provide an overview of current approaches, based on protein and biomaterial engineering that mimic the regulatory functions of ECM to optimize the signaling microenvironment of vascular growth factors.

## Therapeutic Angiogenesis in Regenerative Medicine

Therapeutic angiogenesis aims at restoring blood flow to ischemic tissues by the generation of new vessels. This strategy targets the treatment of ischemic diseases, where endogenous tissue itself is insufficiently perfused, and may improve the rapid vascularization of tissue-engineered grafts, where *in vitro*-generated new tissue is transplanted to repair tissue lost through damage or surgery.

Atherosclerotic cardiovascular disease is the most frequent cause of death in the western world (Norgren et al., 2007; Go et al., 2013). Despite advances in medical and surgical therapy, coronary artery disease (CAD) and peripheral arterial disease (PAD) still have very high mortality and morbidity. No current treatment can stop or revert the process of atherosclerotic vessel obstruction and a considerable number of patients are not suitable candidates for surgical or catheter interventions because of age, co-morbidities, or unfavorable vascular anatomy. Therapeutic angiogenesis aims at restoring the original blood flow in ischemic tissues by delivering factors that control the formation of new vasculature. Although the obstruction is located in the large conductance arteries, expansion of the micro-vascular capillary bed, controlled by angiogenic factors, has been shown to induce the enlargement of upstream collateral arteries through increased shear stress and gap junction-mediated retrograde signaling along vessel walls, thereby effectively producing a biological bypass and restoring downstream perfusion (Rissanen et al., 2005; Pries et al., 2010; Annex, 2013). This strategy represents a very attractive approach for all patients that are not adequately treated by current options.

On the other hand, slow or insufficient vascularization of tissue-engineered grafts is one of the major limiting factors toward their clinical implementation (Scherberich et al., 2010). In fact, while clinical-size tissue grafts can be engineered *in vitro* by culturing autologous progenitors on suitable biomaterial scaffolds, upon *in vivo* implantation the limited diffusion of oxygen and nutrients from surrounding vascular beds allows the engraftment and differentiation of only a thin outer layer. Therefore, in the absence of strategies to provide active vascular ingrowth, tissue-engineered grafts larger than a few millimeters undergo necrosis in the core regions and fail to engraft (Johnson et al., 2007; Rouwkema et al., 2008).

## Angiogenesis and the Extracellular Matrix

**Figure 1:**
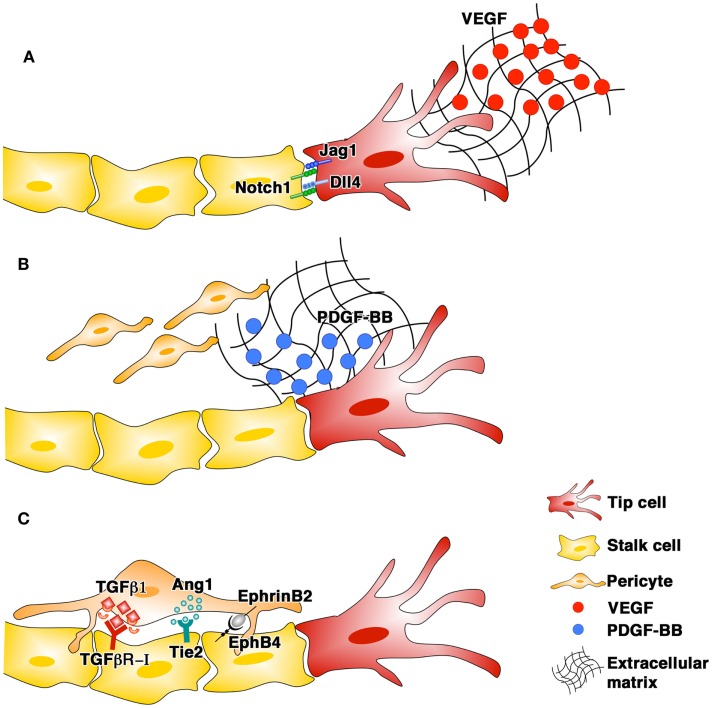
**The phases of blood vessel growth and the main signaling pathways involved: endothelial morphogenesis (A), pericyte recruitment (B), and stabilization (C)**.

Angiogenesis is a complex process in which the growth of normal, stable, and functional vessels is critically dependent on the coordinated interplay in space and time of different cell types and growth factors (GF) (Blau and Banfi, 2001). Activated endothelial cells assemble into new tubular structures (morphogenesis) and subsequently associate with pericytes (maturation). Pericytes provide a variety of regulatory signals, the best characterized of which involve the TGF-β1/TGF-R, Angiopoietin/Tie2, and EphrinB2/EphB4 pathways, leading to endothelial quiescence and new vessel survival independently of further angiogenic stimulation (stabilization) (Figure 1). The spatial localization of angiogenic signals in the extracellular matrix (ECM) plays a fundamental role in ensuring the proper completion of all steps in vascular formation.

Vascular endothelial growth factor-A (VEGF) is the master regulator of both physiological and pathological angiogenesis and is capable, when delivered as a single factor, to start the complex cascade of events leading to new vascular growth (Potente et al., 2011). VEGF exists in three major splicing isoforms, which are composed of 120/121, 164/165, and 188/189 amino acids in mouse and human, respectively, and differ in their affinity for ECM (Park et al., 1993). Studies in transgenic models expressing only one of the major VEGF isoforms showed that their specific ability to bind ECM is critical for proper vascular morphogenesis. VEGF_120_, which does not bind ECM, induces vascular networks with reduced branching and abnormally enlarged diameters, VEGF_188_, which binds ECM strongly, causes opposite defects, with ectopic branching and reduced capillary size, whereas VEGF_164_, which binds ECM with intermediate affinity, is the only isoform capable of inducing physiologically patterned vasculature (Ruhrberg et al., 2002). Remarkably, the combination of VEGF_120_ and VEGF_188_ also leads to normal angiogenesis in the absence of VEGF_164_, showing that a balanced matrix-binding affinity, rather than a specific isoform, is required for proper vascular morphogenesis (Ruhrberg et al., 2002). The formation of microenvironmental VEGF gradients in the tissue matrix guides new capillary sprouting. The first endothelial cells activated by VEGF become specialized tip cells, which sense the VEGF gradient by extending thin filopodial processes and migrate toward its source (Gerhardt et al., 2003). Notch signaling regulates this process, as tip cells upregulate the Notch ligand Delta-like-4 (Dll4) and activate Notch1 in adjacent cells, instructing them to function as stalk cells (Hellstrom et al., 2007), which proliferate to form the main trunk of the new vessel behind the migrating tip (Gerhardt et al., 2003). However, in conditions of pathologically high VEGF, with impaired gradient formation, the alternate pattern of Dll4 expression and Notch activation and the orderly formation of tip and stalk cells are disrupted (Bentley et al., 2008).

Pericytes are recruited to nascent vessels by platelet-derived growth factor-BB (PDGF-BB) produced by activated endothelium. PDGF-BB also binds strongly to the ECM through a carboxy-terminal stretch of positively charged amino acids (Ostman et al., 1991), forming steep gradients. PDGF-BB retention in the matrix is an absolute requirement for proper vascular maturation. In fact, deletion of the retention motif from the endogenous *Pdgfb* gene, leading to free diffusion of the expressed PDGF-BB, causes pericyte detachment from endothelium and defective angiogenesis, with retinal deterioration and sclerosis of renal glomeruli and proteinuria (Lindblom et al., 2003). We recently found that co-delivery of PDGF-BB can normalize the aberrant angiogenesis induced by high and uncontrolled levels of VEGF, through retention of pericytes on remodeling vascular structures. However, the two GF required co-expression at a fixed relative level from a single bicistronic vector to ensure co-localized gradients in the tissue, whereas the same doses from separate vectors or cell populations were not effective (Banfi et al., 2012).

## Limitations of VEGF Delivery for Therapeutic Angiogenesis

All key angiogenic GFs, including VEGF-A, FGF-2, IGF, HGF, PDGF-BB, and TGF-β1, share the common property of binding ECM (Martino and Hubbell, 2010), which is crucially required for their biological function to guide vascular growth toward the hypoxic areas. Matrix interactions of angiogenic GF have profound therapeutic implications and here we will discuss how this affects delivery strategies, with the best-studied factor, VEGF, as a paradigm. For therapeutic purposes, two main parameters need to be controlled in order to ensure both safety and efficacy: dose and duration of stimulation.

Despite encouraging preclinical data and phase I clinical studies, the outcome of placebo-controlled clinical trials of VEGF gene therapy for coronary and peripheral artery disease has been disappointing and clear clinical benefit has yet to be established (Gupta et al., 2009; Giacca and Zacchigna, 2012). Retrospective analyses of clinical trials identified several issues that undermined their efficacy, particularly the difficulty to deliver enough VEGF at safe vector doses into the target tissue to generate sufficient angiogenesis and correct ischemia (Yla-Herttuala et al., 2004; Karvinen and Yla-Herttuala, 2010). Elegant genetic experiments have shown that during development exquisite control of VEGF dose is required, as variations in its levels of expression as small as a 50% reduction (Carmeliet et al., 1996; Ferrara et al., 1996) or a two- to threefold increase (Miquerol et al., 2000) are lethal. Upon delivery of exogenous VEGF to adult tissues, increased vessel permeability causes severe edema and loss of limb in animals (Masaki et al., 2002; Vajanto et al., 2002). Using gene delivery systems such as retrovirally transduced myoblasts (Carmeliet, 2000; Lee et al., 2000), adeno- and adeno-associated viral vectors (Pettersson et al., 2000; Sundberg et al., 2001; Karvinen et al., 2011), and plasmid DNA (Isner et al., 1996; Schwarz et al., 2000), it was shown that uncontrolled VEGF expression induces the growth of vascular tumors (hemangiomas) in skeletal muscle (Springer et al., 1998), myocardium, and other tissues.

However, we found evidence that VEGF does not have an intrinsically steep dose–response curve *in vivo*, but rather that the dose delivered must be controlled at the microenvironmental level (Ozawa et al., 2004; Von Degenfeld et al., 2006; Misteli et al., 2010; Melly et al., 2012; Wolff et al., 2012; Mujagic et al., 2013). In fact, due to the ECM-binding of VEGF, different GF concentrations remain tightly localized after secretion and a few “hotspots” of high expression can cause angioma growth even if the total dose is rather low. Therefore, the same total dose of VEGF can have disparate effects, therapeutic or toxic, depending on whether it is distributed homogeneously in the tissue or not (Banfi et al., 2005).

On the other hand, sufficient duration of VEGF expression for at least about 4 weeks is also critical for newly induced vessels to stabilize and persist (Dor et al., 2002; Ozawa et al., 2004; Tafuro et al., 2009).

Therapeutic challenges for delivering angiogenic GFs include the need to control the microenvironmental distribution of their levels in tissue, which is inherently difficult with direct gene therapy approaches, while recombinant proteins, which could overcome this issue, have too short half-lives *in vivo*. Tremendous work has been done to engineer biomaterials allowing sustained release of angiogenic GFs. In general, the biochemical and biophysical properties of natural or synthetic biomaterials are modified to ensure passive release of embedded GFs at controllable rates. These approaches are covered in a number of excellent reviews (Fischbach and Mooney, 2007; Phelps and Garcia, 2009; Lee et al., 2011; Chu and Wang, 2012). On the other hand, fundamentally different approaches aim at functionalizing biomaterials with GF-binding domains derived from the ECM or directly modifying GFs to specifically bind biomaterial matrices. The next sections will highlight these approaches aimed at controlled presentation of angiogenic GFs in their physiological ECM-bound context to overcome their therapeutic limitations.

## Engineering Biomaterial Matrices to Optimize the Delivery of Angiogenic Growth Factors

**Figure 2:**
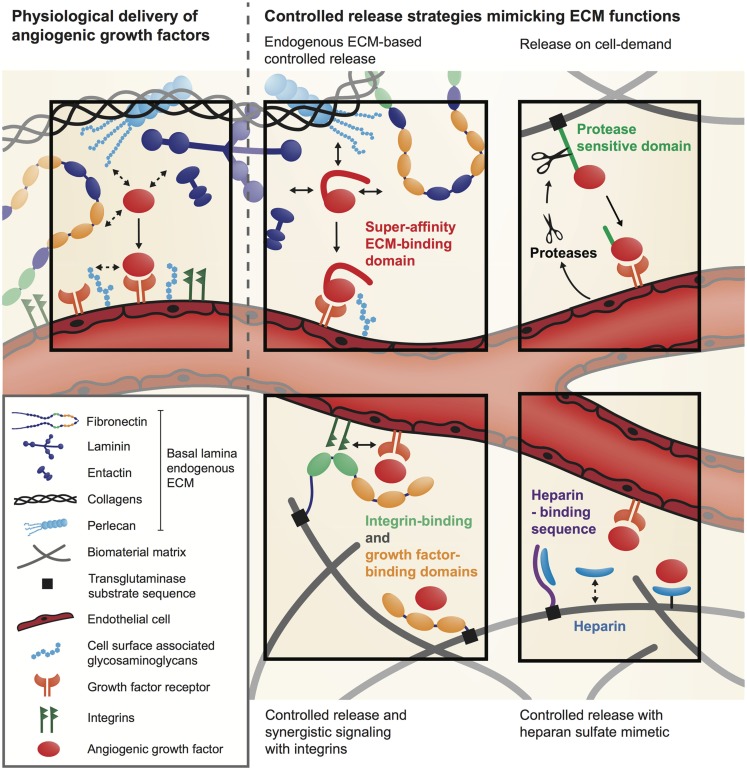
**Delivery systems for angiogenic GFs inspired by the natural GF regulatory function of the ECM**.

Extracellular matrix physiologically orchestrates the activity of angiogenic GFs, regulating their local concentration, partitioning, bioavailability, and signaling. To recapitulate the ECM regulatory functions, an elegant approach is to functionalize biomaterial matrices with specific GF-binding sites derived from the ECM (Figure 2).

### Functionalization of biomaterial matrices with growth factor-binding sites

Most angiogenic GFs have the ability to bind specific sites in the ECM (Schonherr and Hausser, 2000; Schultz and Wysocki, 2009) and they will first interact with the ECM before finding their cell-surface receptor or co-receptor. For example, VEGF-A_165_, FGF-2, and PDGF-BB possess specific interactions with heparan sulfate proteoglycans within the ECM (Capila and Linhardt, 2002; Macri et al., 2007). Moreover, several GF-binding sites have recently been discovered within ECM proteins such as fibronectin (Martino and Hubbell, 2010), fibrinogen (Martino et al., 2013), tenascin C (De Laporte et al., 2013), and vitronectin (Upton et al., 2008). Therefore, once bound to the ECM, angiogenic GFs are released depending on their binding affinity and the action of proteases that specifically cleave ECM or the ECM-binding domains of GFs (Martino et al., 2013, 2014). Consequently, ECM releases signaling molecules at different kinetics and from different locations, which allows a very tight spatio-temporal regulation of angiogenesis (Hynes, 2009; Schultz and Wysocki, 2009). To mimic these GF-binding features, biomaterial matrices have been modified with heparin or heparan sulfate-mimetic molecules that sequester heparin-binding GFs and control their release (Pike et al., 2006; Nillesen et al., 2007; Lee et al., 2011) (Figure 2). For example, synthetic hydrogel films cross-linked with heparin and derivatives of chondroitin sulfate have been used to successfully control the delivery of FGF-2 in a full-thickness excisional wound model in *db/db* diabetic mice and showed acceleration of vascularization (Liu et al., 2007).

Growth factor-binding sites from ECM proteins have also been used to control the presentation of angiogenic factors (Martino and Hubbell, 2010; Martino et al., 2011, 2013) (Figure 2). Remarkably, these binding sites are often promiscuous for multiple GFs and offer the possibility to co-deliver different GFs. For example, fibrin(ogen) has a natural affinity for several angiogenic GFs and fibrin matrices have been successfully used to deliver low doses of FGF-2 and placenta growth factor-2 (PlGF-2) for wound healing in diabetic mice (Martino et al., 2013). Moreover, the GF-binding domain of fibrin(ogen) has been isolated and incorporated in a synthetic PEG-based matrix, which then could sequester GFs similarly to natural fibrin. Interestingly, treatment of wounds in diabetic mice with FGF-2 and PlGF-2 co-delivery through the synthetic matrix induced similar angiogenesis as with GF delivery through fibrin (Martino et al., 2013). Therefore, integrating ECM GF-binding domains into biomaterial matrices allows efficient delivery of low doses of angiogenic GFs. Alternatively, biomaterials were functionalized with a VEGF-binding peptide derived from VEGF-Receptor2 to allow controlled release of VEGF (Impellitteri et al., 2012).

### Optimizing the signaling microenvironment of angiogenic growth factors

Besides controlling GF bioavailability, the ECM also modulates GF signaling through the interplay between GFs, ECM proteins, cell-adhesion receptors, and GF receptors (Giancotti and Tarone, 2003; Hynes, 2009; Kim et al., 2011). For example, the binding of VEGF_165_ to fibronectin forms molecular complexes that induce the formation of clusters between VEGF receptor and integrins (Wijelath et al., 2006; Martino et al., 2011, 2013). Because integrins and GF receptors share several molecules in their signaling machinery, these clusters can considerably enhance signaling (Yamada and Even-Ram, 2002; Comoglio et al., 2003; Martino et al., 2011). Thus, depending on the composition of the ECM surrounding GF receptors, GFs induce a more or less strong signaling. This synergistic signaling between integrins and GF receptors can be exploited to lower the dose of angiogenic GF delivered (Figure 2). For example, a multifunctional recombinant fragment of fibronectin has been engineered to integrate a fibrin-binding sequence, an integrin-binding domain and a GF-binding domain. In a chronic wound model in *db/db* mice, co-delivery of VEGF_165_ and PDGF-BB with this multifunctional fibronectin fragment was able to induce angiogenesis at low doses, while GFs delivered without the fibronectin fragment had no significant effect (Martino et al., 2011).

## Engineering Angiogenic Growth Factors to Interact with Natural Biomaterial Matrices and Endogenous ECM

Instead of modifying biomaterials to enhance their affinity for angiogenic GFs, the factors themselves can be engineered to increase their affinity for biomaterial matrices or for the endogenous ECM present at the delivery site. As a first approach, GFs can be covalently immobilized into a biomaterial matrix using chemical or enzymatic reactions. A variety of chemical conjugation methods have been developed (Zisch et al., 2003; Phelps et al., 2013), but a potential limitation is that angiogenic GFs may lose their activity after coupling. To address this issue, an elegant technique has been developed to covalently cross-link GFs into fibrin (Ehrbar et al., 2004, 2008) or fibrin-mimetic PEG matrices (Ehrbar et al., 2007). GFs are engineered to contain an octapeptide sequence derived from alpha-2-plasmin inhibitor (NQEQVSPL), which is a substrate for the transglutaminase factor XIIIa. Because fibrin and fibrin-mimetic PEG matrices are polymerized by factor XIIIa, the engineered GFs are covalently incorporated into the matrices during the polymerization process (Figure 2). This specific enzymatic cross-linking of GFs into fibrin was demonstrated to be effective to deliver VEGF in various animal models (Ehrbar et al., 2004; Traub et al., 2013; Sacchi et al., 2014). For example, optimized delivery of fibrin-bound VEGF was therapeutically effective both in ischemic hind limb and wound-healing models, by significantly improving angiogenesis, tissue perfusion, and healing rate (Sacchi et al., 2014).

It should be noted that, when GFs are covalently coupled to a biomaterial matrix, their release will depend on matrix degradation and therefore the release rate will depend on the local activity of degrading cells and enzymes. For example, GFs linked to fibrin are released by the action of proteases such as matrix metalloproteinases and plasmin. In order to control the GF release kinetics and couple release to cellular demand, GFs can be engineered to incorporate a protease-sensitive site between the factor sequence and the fibrin-coupling site (Ehrbar et al., 2004; Traub et al., 2013) (Figure 2). Moreover, to fully customize release rates, the fibrin matrix can be further functionalized with different concentrations of the plasmin inhibitor aprotinin, which, in balance with the local inflammatory environment, will determine the specific functional outcome of a specific GF concentration (Sacchi et al., 2014).

Another approach consists of engineering GFs to enhance their affinity for a natural biomaterial matrix such as fibrin, collagen, and endogenous ECM, without covalent coupling. Recently, it has been demonstrated that targeting endogenous ECM can enhance the efficacy of angiogenic GFs even when they are delivered at low doses (Martino et al., 2014). Twenty-five GFs were screened for their binding to six key ECM proteins, namely fibronectin, vitronectin, tenascin C, osteopontin, fibrinogen, and collagen I. The study revealed that PlGF-2 displays a very strong affinity to ECM proteins, especially fibronectin, tenascin C, osteopontin, and fibrinogen, as well as heparan sulfate through its heparin-binding sequence (PlGF-2_123–144_). Using rational protein engineering, PlGF-2_123–144_ has been incorporated into angiogenic GFs that have clinical translation limitations, namely VEGF and PDGF-BB, endowing them with super-affinity for ECM proteins and heparan sulfate (Figure 2). In a wound-healing model in diabetic mice, low doses of PlGF-2_123–144_-fused PDGF-BB and VEGF led to significantly faster wound closure and to more granulation angiogenesis compared to the wild-type factors, both topically and in fibrin. Moreover, one of the critical clinical limitations of VEGF, i.e., its induction of vascular hyperpermeability, was ameliorated through this GF engineering concept (Martino et al., 2014). Once delivered at the target site, VEGF rapidly activates its receptor and induces a burst signaling. In contrast, the number of receptors activated within the same period of time is most likely much lower when delivering VEGF/PlGF-2_123–144_, because the engineered GF is retained within the endogenous ECM. This could help controlling the unwanted effect of VEGF on vascular permeability, which is directly linked to the number of active VEGF receptor molecules. In fact, VEGF/PlGF-2_123–144_ induced only 10% of vascular leakage compared to the same dose of wild-type VEGF, but induced more angiogenesis (Martino et al., 2014).

## Future Directions

Today, the main challenge to overcome, when designing angiogenic therapies based on recombinant GFs, is the control of their local delivery. Moreover, since angiogenesis involves multiple sequential signals, the delivery of multiple GFs may be required to efficiently promote angiogenesis (Borselli et al., 2010; Anderson et al., 2014). For example, systems engineered to reproduce the sequential presentation of the factors controlling the phases of endothelial morphogenesis and vessel maturation may promote a more physiological vascularization (Richardson et al., 2001; Hao et al., 2007; Ruvinov et al., 2011; Brudno et al., 2013).

In conclusion, understanding how GFs are presented by the ECM during physiological blood vessel growth is critical to develop efficient and safe GF delivery platforms. Especially, approaches mimicking the GF regulatory function of the ECM with a relatively simple regulatory path are expected to be particularly relevant toward clinical translation.

## Conflict of Interest Statement

The fibrin gel immobilization scheme presented is the subject of patents upon which Jeffrey A. Hubbell is named as inventor and has been licensed by a company in which Jeffrey A. Hubbell is a shareholder. The growth factor engineering approach to enhance ECM protein affinity is the subject of a patent application upon which Jeffrey A. Hubbell, Mikaël M. Martino, and Priscilla S. Briquez are named as inventors and has been licensed to a company in which Jeffrey A. Hubbell is a shareholder. The other co-authors declare that the research was conducted in the absence of any commercial or financial relationships that could be construed as a potential conflict of interest.

## References

[B1] Anderson E. M. Kwee B. J. Lewin S. A. Raimondo T. Mehta M. Mooney D. J. (2014). Local delivery of VEGF and SDF enhances endothelial progenitor cell recruitment and resultant recovery from ischemia. Tissue Eng. Part A 10.1089/ten.TEA.2014.0508 25434326 PMC4394875

[B2] Annex B. H. (2013). Therapeutic angiogenesis for critical limb ischaemia. Nat. Rev. Cardiol. 10, 387–396. 10.1038/nrcardio.2013.70 23670612

[B3] Banfi A. Von Degenfeld G. Blau H. M. (2005). Critical role of microenvironmental factors in angiogenesis. Curr. Atheroscler. Rep. 7, 227–234. 10.1007/s11883-005-0011-7 15811258

[B4] Banfi A. Von Degenfeld G. Gianni-Barrera R. Reginato S. Merchant M. J. McDonald D. M. (2012). Therapeutic angiogenesis due to balanced single-vector delivery of VEGF and PDGF-BB. FASEB J. 26, 2486–2497. 10.1096/fj.11-197400 22391130 PMC3360154

[B5] Bentley K. Gerhardt H. Bates P. A. (2008). Agent-based simulation of notch-mediated tip cell selection in angiogenic sprout initialisation. J. Theor. Biol. 250, 25–36. 10.1016/j.jtbi.2007.09.015 18028963

[B6] Blau H. M. Banfi A. (2001). The well-tempered vessel. Nat. Med. 7, 532–534 10.1038/87850 11329048

[B7] Borselli C. Storrie H. Benesch-Lee F. Shvartsman D. Cezar C. Lichtman J. W. (2010). Functional muscle regeneration with combined delivery of angiogenesis and myogenesis factors. Proc. Natl. Acad. Sci. U.S.A. 107, 3287–3292. 10.1073/pnas.0903875106 19966309 PMC2840452

[B8] Brudno Y. Ennett-Shepard A. B. Chen R. R. Aizenberg M. Mooney D. J. (2013). Enhancing microvascular formation and vessel maturation through temporal control over multiple pro-angiogenic and pro-maturation factors. Biomaterials 34, 9201–9209. 10.1016/j.biomaterials.2013.08.007 23972477 PMC3811005

[B9] Capila I. Linhardt R. J. (2002). Heparin-protein interactions. Angew. Chem. Int. Ed. Engl. 41, 390–412 10.1002/1521-3773(20020201)41:3<390::AID-ANIE390>3.0.CO;2-B 12491369

[B10] Carmeliet P. (2000). VEGF gene therapy: stimulating angiogenesis or angioma-genesis? Nat. Med. 6, 1102–1103 10.1038/80430 11017137

[B11] Carmeliet P. Ferreira V. Breier G. Pollefeyt S. Kieckens L. Gertsenstein M. (1996). Abnormal blood vessel development and lethality in embryos lacking a single VEGF allele. Nature 380, 435–439. 10.1038/380435a0 8602241

[B12] Chu H. Wang Y. (2012). Therapeutic angiogenesis: controlled delivery of angiogenic factors. Ther. Deliv. 3, 693–714. 10.4155/tde.12.50 22838066 PMC3564557

[B13] Comoglio P. M. Boccaccio C. Trusolino L. (2003). Interactions between growth factor receptors and adhesion molecules: breaking the rules. Curr. Opin. Cell Biol. 15, 565–571. 10.1016/S0955-0674(03)00096-6 14519391

[B14] De Laporte L. Rice J. J. Tortelli F. Hubbell J. A. (2013). Tenascin C promiscuously binds growth factors via its fifth fibronectin type III-like domain. PLoS ONE 8:e62076. 10.1371/journal.pone.0062076 23637968 PMC3630135

[B15] Dor Y. Djonov V. Abramovitch R. Itin A. Fishman G. I. Carmeliet P. (2002). Conditional switching of VEGF provides new insights into adult neovascularization and pro-angiogenic therapy. EMBO J. 21, 1939–1947. 10.1093/emboj/21.8.1939 11953313 PMC125962

[B16] Ehrbar M. Djonov V. G. Schnell C. Tschanz S. A. Martiny-Baron G. Schenk U. (2004). Cell-demanded liberation of VEGF121 from fibrin implants induces local and controlled blood vessel growth. Circ. Res. 94, 1124–1132. 10.1161/01.RES.0000126411.29641.08 15044320

[B17] Ehrbar M. Rizzi S. C. Hlushchuk R. Djonov V. Zisch A. H. Hubbell J. A. (2007). Enzymatic formation of modular cell-instructive fibrin analogs for tissue engineering. Biomaterials 28, 3856–3866. 10.1016/j.biomaterials.2007.03.027 17568666

[B18] Ehrbar M. Zeisberger S. M. Raeber G. P. Hubbell J. A. Schnell C. Zisch A. H. (2008). The role of actively released fibrin-conjugated VEGF for VEGF receptor 2 gene activation and the enhancement of angiogenesis. Biomaterials 29, 1720–1729. 10.1016/j.biomaterials.2007.12.002 18155761

[B19] Ferrara N. Carver-Moore K. Chen H. Dowd M. Lu L. O’Shea K. S. (1996). Heterozygous embryonic lethality induced by targeted inactivation of the VEGF gene. Nature 380, 439–442. 10.1038/380439a0 8602242

[B20] Fischbach C. Mooney D. J. (2007). Polymers for pro- and anti-angiogenic therapy. Biomaterials 28, 2069–2076. 10.1016/j.biomaterials.2006.12.029 17254631

[B21] Gerhardt H. Golding M. Fruttiger M. Ruhrberg C. Lundkvist A. Abramsson A. (2003). VEGF guides angiogenic sprouting utilizing endothelial tip cell filopodia. J. Cell Biol. 161, 1163–1177. 10.1083/jcb.200302047 12810700 PMC2172999

[B22] Giacca M. Zacchigna S. (2012). VEGF gene therapy: therapeutic angiogenesis in the clinic and beyond. Gene Ther. 19, 622–629. 10.1038/gt.2012.17 22378343

[B23] Giancotti F. G. Tarone G. (2003). Positional control of cell fate through joint integrin/receptor protein kinase signaling. Annu. Rev. Cell Dev. Biol. 19, 173–206. 10.1146/annurev.cellbio.19.031103.133334 14570568

[B24] Go A. S. Mozaffarian D. Roger V. L. Benjamin E. J. Berry J. D. Borden W. B. (2013). Heart disease and stroke statistics – 2013 update: a report from the American Heart Association. Circulation 127, e6–e245 10.1161/CIR.0b013e31828124ad 23239837 PMC5408511

[B25] Gupta R. Tongers J. Losordo D. W. (2009). Human studies of angiogenic gene therapy. Circ. Res. 105, 724–736 10.1161/CIRCRESAHA.109.200386 19815827 PMC2770893

[B26] Hao X. Silva E. A. Mansson-Broberg A. Grinnemo K. H. Siddiqui A. J. Dellgren G. (2007). Angiogenic effects of sequential release of VEGF-A165 and PDGF-BB with alginate hydrogels after myocardial infarction. Cardiovasc. Res. 75, 178–185. 10.1016/j.cardiores.2007.03.028 17481597

[B27] Hellstrom M. Phng L. K. Hofmann J. J. Wallgard E. Coultas L. Lindblom P. (2007). Dll4 signalling through Notch1 regulates formation of tip cells during angiogenesis. Nature 445, 776–780. 10.1038/nature05571 17259973

[B28] Hynes R. O. (2009). The extracellular matrix: not just pretty fibrils. Science 326, 1216–1219. 10.1126/science.1176009 19965464 PMC3536535

[B29] Impellitteri N. A. Toepke M. W. Lan Levengood S. K. Murphy W. L. (2012). Specific VEGF sequestering and release using peptide-functionalized hydrogel microspheres. Biomaterials 33, 3475–3484. 10.1016/j.biomaterials.2012.01.032 22322198 PMC3307527

[B30] Isner J. M. Pieczek A. Schainfeld R. Blair R. Haley L. Asahara T. (1996). Clinical evidence of angiogenesis after arterial gene transfer of phVEGF165 in patient with ischaemic limb. Lancet 348, 370–374. 10.1016/S0140-6736(96)03361-2 8709735

[B31] Johnson P. C. Mikos A. G. Fisher J. P. Jansen J. A. (2007). Strategic directions in tissue engineering. Tissue Eng. 13, 2827–2837. 10.1089/ten.2007.0335 18052823

[B32] Karvinen H. Pasanen E. Rissanen T. T. Korpisalo P. Vahakangas E. Jazwa A. (2011). Long-term VEGF-A expression promotes aberrant angiogenesis and fibrosis in skeletal muscle. Gene Ther. 18, 1166–1172. 10.1038/gt.2011.66 21562595

[B33] Karvinen H. Yla-Herttuala S. (2010). New aspects in vascular gene therapy. Curr. Opin. Pharmacol. 10, 208–211. 10.1016/j.coph.2010.01.004 20163988

[B34] Kim S. H. Turnbull J. Guimond S. (2011). Extracellular matrix and cell signalling: the dynamic cooperation of integrin, proteoglycan and growth factor receptor. J. Endocrinol. 209, 139–151. 10.1530/JOE-10-0377 21307119

[B35] Lee K. Silva E. A. Mooney D. J. (2011). Growth factor delivery-based tissue engineering: general approaches and a review of recent developments. J. R. Soc. Interface 8, 153–170. 10.1098/rsif.2010.0223 20719768 PMC3033020

[B36] Lee R. J. Springer M. L. Blanco-Bose W. E. Shaw R. Ursell P. C. Blau H. M. (2000). VEGF gene delivery to myocardium: deleterious effects of unregulated expression. Circulation 102, 898–901. 10.1161/01.CIR.102.8.898 10952959

[B37] Lindblom P. Gerhardt H. Liebner S. Abramsson A. Enge M. Hellstrom M. (2003). Endothelial PDGF-B retention is required for proper investment of pericytes in the microvessel wall. Genes Dev. 17, 1835–1840. 10.1101/gad.266803 12897053 PMC196228

[B38] Liu Y. Cai S. Shu X. Z. Shelby J. Prestwich G. D. (2007). Release of basic fibroblast growth factor from a crosslinked glycosaminoglycan hydrogel promotes wound healing. Wound Repair Regen. 15, 245–251. 10.1111/j.1524-475X.2007.00211.x 17352757

[B39] Macri L. Silverstein D. Clark R. A. (2007). Growth factor binding to the pericellular matrix and its importance in tissue engineering. Adv. Drug Deliv. Rev. 59, 1366–1381. 10.1016/j.addr.2007.08.015 17916397

[B40] Martino M. M. Briquez P. S. Guc E. Tortelli F. Kilarski W. W. Metzger S. (2014). Growth factors engineered for super-affinity to the extracellular matrix enhance tissue healing. Science 343, 885–888. 10.1126/science.1247663 24558160

[B41] Martino M. M. Briquez P. S. Ranga A. Lutolf M. P. Hubbell J. A. (2013). Heparin-binding domain of fibrin(ogen) binds growth factors and promotes tissue repair when incorporated within a synthetic matrix. Proc. Natl. Acad. Sci. U.S.A. 110, 4563–4568. 10.1073/pnas.1221602110 23487783 PMC3607046

[B42] Martino M. M. Hubbell J. A. (2010). The 12th-14th type III repeats of fibronectin function as a highly promiscuous growth factor-binding domain. FASEB J. 24, 4711–4721. 10.1096/fj.09-151282 20671107

[B43] Martino M. M. Tortelli F. Mochizuki M. Traub S. Ben-David D. Kuhn G. A. (2011). Engineering the growth factor microenvironment with fibronectin domains to promote wound and bone tissue healing. Sci. Transl. Med. 3, 100ra189. 10.1126/scitranslmed.3002614 21918106

[B44] Masaki I. Yonemitsu Y. Yamashita A. Sata S. Tanii M. Komori K. (2002). Angiogenic gene therapy for experimental critical limb ischemia: acceleration of limb loss by overexpression of vascular endothelial growth factor 165 but not of fibroblast growth factor-2. Circ. Res. 90, 966–973. 10.1161/01.RES.0000019540.41697.60 12016262

[B45] Melly L. F. Marsano A. Frobert A. Boccardo S. Helmrich U. Heberer M. (2012). Controlled angiogenesis in the heart by cell-based expression of specific vascular endothelial growth factor levels. Hum. Gene Ther. Methods 23, 346–356. 10.1089/hgtb.2012.032 23075102 PMC4015223

[B46] Miquerol L. Langille B. L. Nagy A. (2000). Embryonic development is disrupted by modest increases in vascular endothelial growth factor gene expression. Development 127, 3941–3946. 10952892 10.1242/dev.127.18.3941

[B47] Misteli H. Wolff T. Fuglistaler P. Gianni-Barrera R. Gurke L. Heberer M. (2010). High-throughput flow cytometry purification of transduced progenitors expressing defined levels of vascular endothelial growth factor induces controlled angiogenesis in vivo. Stem Cells 28, 611–619. 10.1002/stem.291 20039367

[B48] Mujagic E. Gianni-Barrera R. Trani M. Patel A. Gurke L. Heberer M. (2013). Induction of aberrant vascular growth, but not of normal angiogenesis, by cell-based expression of different doses of human and mouse VEGF is species-dependent. Hum. Gene Ther. Methods 24, 28–37. 10.1089/hgtb.2012.197 23360398 PMC4015081

[B49] Nillesen S. T. Geutjes P. J. Wismans R. Schalkwijk J. Daamen W. F. Van Kuppevelt T. H. (2007). Increased angiogenesis and blood vessel maturation in acellular collagen-heparin scaffolds containing both FGF2 and VEGF. Biomaterials 28, 1123–1131. 10.1016/j.biomaterials.2006.10.029 17113636

[B50] Norgren L. Hiatt W. R. Dormandy J. A. Nehler M. R. Harris K. A. Fowkes F. G. (2007). Inter-society consensus for the management of peripheral arterial disease (TASC II). J. Vasc. Surg. 45(Suppl. S), S5–S67 10.1016/j.jvs.2006.12.037 17223489

[B51] Ostman A. Andersson M. Betsholtz C. Westermark B. Heldin C. H. (1991). Identification of a cell retention signal in the B-chain of platelet-derived growth factor and in the long splice version of the A-chain. Cell Regul. 2, 503–512. 1782212 10.1091/mbc.2.7.503 PMC361840

[B52] Ozawa C. R. Banfi A. Glazer N. L. Thurston G. Springer M. L. Kraft P. E. (2004). Microenvironmental VEGF concentration, not total dose, determines a threshold between normal and aberrant angiogenesis. J. Clin. Invest. 113, 516–527. 10.1172/JCI18420 14966561 PMC338257

[B53] Park J. E. Keller G. A. Ferrara N. (1993). The vascular endothelial growth factor (VEGF) isoforms: differential deposition into the subepithelial extracellular matrix and bioactivity of extracellular matrix-bound VEGF. Mol. Biol. Cell 4, 1317–1326. 10.1091/mbc.4.12.1317 8167412 PMC275767

[B54] Pettersson A. Nagy J. A. Brown L. F. Sundberg C. Morgan E. Jungles S. (2000). Heterogeneity of the angiogenic response induced in different normal adult tissues by vascular permeability factor/vascular endothelial growth factor. Lab. Invest. 80, 99–115. 10.1038/labinvest.3780013 10653008

[B55] Phelps E. A. Garcia A. J. (2009). Update on therapeutic vascularization strategies. Regen. Med. 4, 65–80. 10.2217/17460751.4.1.65 19105617 PMC2644334

[B56] Phelps E. A. Headen D. M. Taylor W. R. Thule P. M. Garcia A. J. (2013). Vasculogenic bio-synthetic hydrogel for enhancement of pancreatic islet engraftment and function in type 1 diabetes. Biomaterials 34, 4602–4611. 10.1016/j.biomaterials.2013.03.012 23541111 PMC3628538

[B57] Pike D. B. Cai S. Pomraning K. R. Firpo M. A. Fisher R. J. Shu X. Z. (2006). Heparin-regulated release of growth factors in vitro and angiogenic response in vivo to implanted hyaluronan hydrogels containing VEGF and bFGF. Biomaterials 27, 5242–5251. 10.1016/j.biomaterials.2006.05.018 16806456

[B58] Potente M. Gerhardt H. Carmeliet P. (2011). Basic and therapeutic aspects of angiogenesis. Cell 146, 873–887 10.1016/j.cell.2011.08.039 21925313

[B59] Pries A. R. Höpfner M. Le Noble F. Dewhirst M. W. Secomb T. W. (2010). The shunt problem: control of functional shunting in normal and tumour vasculature. Nat. Rev. Cancer 10, 587–593. 10.1038/nrc2895 20631803 PMC3109666

[B60] Richardson T. P. Peters M. C. Ennett A. B. Mooney D. J. (2001). Polymeric system for dual growth factor delivery. Nat. Biotechnol. 19, 1029–1034. 10.1038/nbt1101-1029 11689847

[B61] Rissanen T. T. Korpisalo P. Markkanen J. E. Liimatainen T. Ordén M. R. Kholová I. (2005). Blood flow remodels growing vasculature during vascular endothelial growth factor gene therapy and determines between capillary arterialization and sprouting angiogenesis. Circulation 112, 3937–3946. 10.1161/CIRCULATIONAHA.105.543124 16344386

[B62] Rouwkema J. Rivron N. C. Van Blitterswijk C. A. (2008). Vascularization in tissue engineering. Trends Biotechnol. 26, 434–441 10.1016/j.tibtech.2008.04.009 18585808

[B63] Ruhrberg C. Gerhardt H. Golding M. Watson R. Ioannidou S. Fujisawa H. (2002). Spatially restricted patterning cues provided by heparin-binding VEGF-A control blood vessel branching morphogenesis. Genes Dev. 16, 2684–2698. 10.1101/gad.242002 12381667 PMC187458

[B64] Ruvinov E. Leor J. Cohen S. (2011). The promotion of myocardial repair by the sequential delivery of IGF-1 and HGF from an injectable alginate biomaterial in a model of acute myocardial infarction. Biomaterials 32, 565–578. 10.1016/j.biomaterials.2010.08.097 20889201

[B65] Sacchi V. Mittermayr R. Hartinger J. Martino M. M. Lorentz K. M. Wolbank S. (2014). Long-lasting fibrin matrices ensure stable and functional angiogenesis by highly tunable, sustained delivery of recombinant VEGF164. Proc. Natl. Acad. Sci. U.S.A. 111, 6952–6957. 10.1073/pnas.1404605111 24778233 PMC4024904

[B66] Scherberich A. Muller A. M. Schafer D. J. Banfi A. Martin I. (2010). Adipose tissue-derived progenitors for engineering osteogenic and vasculogenic grafts. J. Cell. Physiol. 225, 348–353. 10.1002/jcp.22313 20626000

[B67] Schonherr E. Hausser H. J. (2000). Extracellular matrix and cytokines: a functional unit. Dev. Immunol. 7, 89–101. 10.1155/2000/31748 11097204 PMC2276054

[B68] Schultz G. S. Wysocki A. (2009). Interactions between extracellular matrix and growth factors in wound healing. Wound Repair Regen. 17, 153–162 10.1111/j.1524-475X.2009.00466.x 19320882

[B69] Schwarz E. R. Speakman M. T. Patterson M. Hale S. S. Isner J. M. Kedes L. H. (2000). Evaluation of the effects of intramyocardial injection of DNA expressing vascular endothelial growth factor (VEGF) in a myocardial infarction model in the rat – angiogenesis and angioma formation. J. Am. Coll. Cardiol. 35, 1323–1330. 10.1016/S0735-1097(00)00522-2 10758976

[B70] Springer M. L. Chen A. S. Kraft P. E. Bednarski M. Blau H. M. (1998). VEGF gene delivery to muscle: potential role for vasculogenesis in adults. Mol. Cell 2, 549–558. 10.1016/S1097-2765(00)80154-9 9844628

[B71] Sundberg C. Nagy J. A. Brown L. F. Feng D. Eckelhoefer I. A. Manseau E. J. (2001). Glomeruloid microvascular proliferation follows adenoviral vascular permeability factor/vascular endothelial growth factor- 164 gene delivery. Am. J. Pathol. 158, 1145–1160. 10.1016/S0002-9440(10)64062-X 11238063 PMC1850349

[B72] Tafuro S. Ayuso E. Zacchigna S. Zentilin L. Moimas S. Dore F. (2009). Inducible adeno-associated virus vectors promote functional angiogenesis in adult organisms via regulated vascular endothelial growth factor expression. Cardiovasc. Res. 83, 663–671. 10.1093/cvr/cvp152 19443424

[B73] Traub S. Morgner J. Martino M. M. Honing S. Swartz M. A. Wickstrom S. A. (2013). The promotion of endothelial cell attachment and spreading using FNIII10 fused to VEGF-A165. Biomaterials 34, 5958–5968. 10.1016/j.biomaterials.2013.04.050 23683723

[B74] Upton Z. Cuttle L. Noble A. Kempf M. Topping G. Malda J. (2008). Vitronectin: growth factor complexes hold potential as a wound therapy approach. J. Invest. Dermatol. 128, 1535–1544. 10.1038/sj.jid.5701148 18200066

[B75] Vajanto I. Rissanen T. T. Rutanen J. Hiltunen M. O. Tuomisto T. T. Arve K. (2002). Evaluation of angiogenesis and side effects in ischemic rabbit hindlimbs after intramuscular injection of adenoviral vectors encoding VEGF and LacZ. J. Gene Med. 4, 371–380. 10.1002/jgm.287 12124979

[B76] Von Degenfeld G. Banfi A. Springer M. L. Wagner R. A. Jacobi J. Ozawa C. R. (2006). Microenvironmental VEGF distribution is critical for stable and functional vessel growth in ischemia. FASEB J. 20, 2657–2659. 10.1096/fj.06-6568fje 17095533

[B77] Wijelath E. S. Rahman S. Namekata M. Murray J. Nishimura T. Mostafavi-Pour Z. (2006). Heparin-II domain of fibronectin is a vascular endothelial growth factor-binding domain: enhancement of VEGF biological activity by a singular growth factor/matrix protein synergism. Circ. Res. 99, 853–860. 10.1161/01.RES.0000246849.17887.66 17008606 PMC3175430

[B78] Wolff T. Mujagic E. Gianni-Barrera R. Fueglistaler P. Helmrich U. Misteli H. (2012). FACS-purified myoblasts producing controlled VEGF levels induce safe and stable angiogenesis in chronic hind limb ischemia. J. Cell. Mol. Med. 16, 107–117. 10.1111/j.1582-4934.2011.01308.x 21418520 PMC3823097

[B79] Yamada K. M. Even-Ram S. (2002). Integrin regulation of growth factor receptors. Nat. Cell Biol. 4, E75–E76 10.1038/ncb0402-e75 11944037

[B80] Yla-Herttuala S. Markkanen J. E. Rissanen T. T. (2004). Gene therapy for ischemic cardiovascular diseases: some lessons learned from the first clinical trials. Trends Cardiovasc. Med. 14, 295–300. 10.1016/j.tcm.2004.09.001 15596105

[B81] Zisch A. H. Lutolf M. P. Ehrbar M. Raeber G. P. Rizzi S. C. Davies N. (2003). Cell-demanded release of VEGF from synthetic, biointeractive cell ingrowth matrices for vascularized tissue growth. FASEB J. 17, 2260–2262. 10.1096/fj.02-1041fje 14563693

